# TIF-IA-Dependent Regulation of Ribosome Synthesis in *Drosophila* Muscle Is Required to Maintain Systemic Insulin Signaling and Larval Growth

**DOI:** 10.1371/journal.pgen.1004750

**Published:** 2014-10-30

**Authors:** Abhishek Ghosh, Elizabeth J. Rideout, Savraj S. Grewal

**Affiliations:** Department of Biochemistry and Molecular Biology, and Clark H. Smith Brain Tumour Centre, Southern Alberta Cancer Research Institute, University of Calgary, Health Research Innovation Center, Calgary, Alberta, Canada; The University of North Carolina at Chapel Hill, United States of America

## Abstract

The conserved TOR kinase signaling network links nutrient availability to cell, tissue and body growth in animals. One important growth-regulatory target of TOR signaling is ribosome biogenesis. Studies in yeast and mammalian cell culture have described how TOR controls rRNA synthesis—a limiting step in ribosome biogenesis—via the RNA Polymerase I transcription factor TIF-IA. However, the contribution of TOR-dependent ribosome synthesis to tissue and body growth in animals is less clear. Here we show in *Drosophila* larvae that ribosome synthesis in muscle is required non-autonomously to maintain normal body growth and development. We find that amino acid starvation and TOR inhibition lead to reduced levels of TIF-IA, and decreased rRNA synthesis in larval muscle. When we mimic this decrease in muscle ribosome synthesis using RNAi-mediated knockdown of TIF-IA, we observe delayed larval development and reduced body growth. This reduction in growth is caused by lowered systemic insulin signaling via two endocrine responses: reduced expression of *Drosophila* insulin-like peptides (dILPs) from the brain and increased expression of Imp-L2—a secreted factor that binds and inhibits dILP activity—from muscle. We also observed that maintaining TIF-IA levels in muscle could partially reverse the starvation-mediated suppression of systemic insulin signaling. Finally, we show that activation of TOR specifically in muscle can increase overall body size and this effect requires TIF-IA function. These data suggest that muscle ribosome synthesis functions as a nutrient-dependent checkpoint for overall body growth: in nutrient rich conditions, TOR is required to maintain levels of TIF-IA and ribosome synthesis to promote high levels of systemic insulin, but under conditions of starvation stress, reduced muscle ribosome synthesis triggers an endocrine response that limits systemic insulin signaling to restrict growth and maintain homeostasis.

## Author Summary

All animals need adequate nutrition to grow and develop. Studies in tissue culture and model organisms have identified the TOR kinase signaling pathway as a key nutrient-dependent regulator of growth. Under nutrient rich conditions, TOR kinase is active and stimulates metabolic processes that drive growth. Under nutrient poor conditions, TOR is inhibited and animals alter their metabolism to maintain homeostasis and survival. Here we use *Drosophila* larvae to identify a role for ribosome synthesis—a key metabolic process—in mediating nutrient and TOR effects on body growth. In particular, we show that ribosome synthesis specifically in larval muscle is necessary to maintain organismal growth. We find that inhibition of muscle ribosome synthesis leads to reduced systemic insulin-like growth factor signaling via two endocrine responses—decreased expression of brain derived *Drosophila* insulin-like peptides (dILPs) and increased expression of Imp-L2, an inhibitor of insulin signaling. As a result of these effects, body growth is reduced and larval development is delayed. These findings suggest that control of ribosome synthesis, and hence protein synthesis, in specific tissues can exert control on overall body growth.

## Introduction

Nutrient availability is a critical determinant of cell, tissue and body growth in developing animals. Nearly two decades of research has identified the Target-of-Rapamycin (TOR) kinase signaling pathway as a major nutrient-responsive growth pathway in eukaryotes [1], [2]. TOR functions in two distinct complexes – TORC1 and TORC2 – and it is TOR kinase activity specifically within TORC1 that has been established as a growth driver. A complex intracellular signaling network activates TOR kinase activity within TORC1 in response to availability of extracellular nutrients such as amino acids and glucose. TORC1, in turn, stimulates many cell metabolic processes that drive growth and proliferation [2], [3]. In contrast, when nutrients are limiting, TORC1 activity is inhibited and cells switch their metabolism to promote homeostasis and survival during starvation conditions.

One important metabolic target of nutrient/TOR signaling in the control of growth is ribosome biogenesis [4]–[10]. A limiting step of ribosome synthesis is the RNA Polymerase (Pol I)-dependent transcription of ribosomal RNA (rRNA). Studies predominantly in yeast and mammalian cell culture have described mechanisms by which TOR promotes rRNA synthesis [4], [5], [7]–[13]. One target of TOR signaling emerging from these studies is the Pol I-specific transcription factor Transcription Initiation Factor-IA (TIF-IA). TIF-IA associates with Pol I and recruits it to rDNA genes to initiate transcription [5], [6], [9], [14]–[16]. This function of TIF-IA is stimulated by nutrient-dependent activation of TOR, and a handful of reports have proposed mechanisms involving TOR-dependent changes in TIF-IA phosphorylation, levels or localization to rDNA genes [9], [17]. These effects may also involve TOR functioning directly at nucleolar rDNA genes [13]. While these studies provide a molecular basis for understanding how nutrients and TOR control Pol I and rRNA synthesis in cells, the contribution of rRNA and ribosome synthesis to tissue and body growth in developing animals is not as clear.

Genetic studies in model organisms, most notably *Drosophila*, have provided most detail into how nutrient/TOR signaling controls tissue and body growth. During the four-day *Drosophila* larval period, animals increase in mass almost 200-fold [18]. This dramatic growth is nutrition-dependent and mostly occurs in non-dividing polyploid cells that make up the bulk of the larval organs. TOR signaling is central to this size control and functions by coupling dietary protein to growth [19]–[22]. Loss of TOR function in cells or tissues leads to a reduction in cell size or tissue mass, whereas TOR over-activation leads to increased cell and tissue growth [19]–[22].

TOR activity in specific tissues can also influence overall body size through non-autonomous endocrine or systemic effects [23], [24]. An example is the role of TOR in the larval fat body [25], [26]. When dietary proteins are abundant, amino acid uptake into fat body cells stimulates TOR activity. This triggers release of a fat-to-brain secreted signal that promotes the production and release of *Drosophila* insulin-like peptides (dILPs) from neurosecretory cells in the brain [25], [26]. These dILPs then circulate throughout the animal and promote growth in all larval tissues via a conserved insulin receptor/PI3K/Akt signaling pathway [27]. In contrast, when larvae are starved, TOR signaling in the fat body is suppressed leading to reduced circulating dILP levels, and decreased insulin signaling and growth. In this way, TOR activity in the fat body links nutrition to larval growth and development. TOR activity in larval muscle has also been reported to exert systemic effects to promote overall body growth and development [28]. This ability of TOR activity in specific tissues to control whole body metabolism and growth is an emerging theme in both mouse and fly genetic studies [23], [24], [29], [30], and emphasizes the importance of non-autonomous mechanisms in the control of body growth.

In this paper, we describe our ongoing work exploring the role for rRNA synthesis in controlling tissue and body growth in larvae. We find that the nutrient-dependent TOR pathway is required to maintain TIF-IA mRNA and protein levels in larval tissues, especially the muscle, during development. We also find that TIF-IA-dependent ribosome synthesis is required in muscle to maintain systemic insulin signaling and promote normal body growth and development, and loss of TIF-IA in muscle blocks the body growth-promoting effects of TOR signaling. This work emphasizes the importance of non-autonomous, tissue-specific effects of ribosome synthesis on endocrine signaling and body growth during development.

## Results

### Nutrition/TOR signaling maintains TIF-IA mRNA and protein levels in larvae

**Figure 1:**
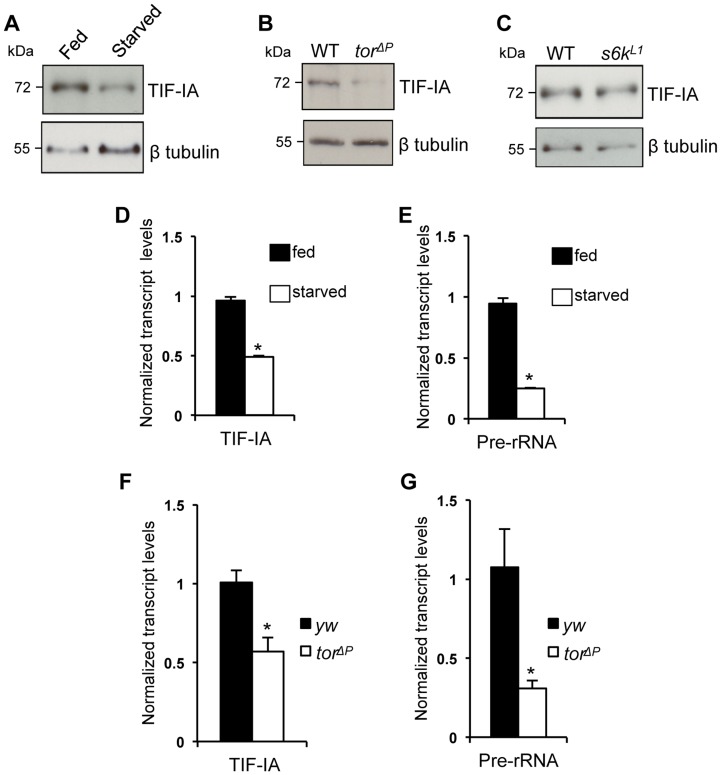
Nutrition-TOR signaling maintains TIF-IA mRNA and protein levels in larvae. (A) Immunoblot indicates TIF-IA protein levels were reduced in 24 hr starved larvae compared to fed larvae. (B) Immunoblot indicates TIF-IA protein levels were reduced in *tor^ΔP^* larvae compared to wild-type (WT) larvae, at 72 hr AEL. (C) Immunoblot indicates TIF-IA protein levels were unchanged between WT and s6k null (*s6k^L1^*) larvae, at 72 hr AEL. In all immunoblots, β tubulin levels indicate loading control. (D) qPCR indicates *TIF-IA* mRNA levels were reduced in 24 hr starved larvae compared to fed larvae. Data normalized to *β tubulin*. (^*^ *P* = 3.46×10^−6^, Student's t-test). (E) qPCR indicates *pre-rRNA* levels were reduced in 24 hr starved larvae compared to fed larvae, at 72 hr AEL. Data normalized to *β tubulin*. (^*^ *P* = 4.47×10^−5^, Student's t-test). (F) qPCR indicates *TIF-IA* mRNA levels were reduced in *tor* larvae compared to *yw* (control) larvae, at 72 hr AEL. Data normalized to *actin*. (^*^ *P* = 0.01, Student's t-test). (G) qPCR indicates *pre-rRNA* levels were reduced in *tor^ΔP^* larvae compared to *yw* (control) larvae, at 72 hr AEL. Data normalized to *actin*. (^*^ *P* = 0.04, Student's t-test). All error bars indicate SEM.

In previous work, we showed that the nutrient/TOR pathway controls rRNA synthesis in developing larvae and that TOR signaling promotes TIF-IA recruitment to rDNA genes [6]. Here, we examined whether TOR signaling may function by controlling TIF-IA levels. Deprivation of dietary protein leads to reduced TOR signaling and decreased rRNA synthesis in larvae. We found that under protein starvation conditions (induced by transferring larvae to a sucrose-only diet), TIF-IA protein levels were reduced compared to fully fed controls (Figure 1A). We also found that TIF-IA protein levels were also reduced in *tor* null mutant (*tor^ΔP^*) larvae compared to wild-type controls (Figure 1B). TOR can promote growth in part via its downstream effector kinase, ribosomal protein S6 kinase (S6K) [31]. However, we found that that TIF-IA protein levels were unaltered in *s6k* mutant (*s6k^L1^*) larvae compared to wild-type (Figure 1C). These results prompted us to examine TIF-IA mRNA levels. We found that both starved larvae and *tor^ΔP^* mutant larvae had reduced levels of both TIF-IA mRNA (Figure 1D, F) and pre-rRNA (Figure 1E, G) consistent with a reduction in synthesis of rRNA and hence ribosomes. Thus, during larval development nutrient/TOR signaling is required to maintain appropriate levels of TIF-IA mRNA and protein.

### TIF-IA function is required in muscle to maintain overall body growth and development

**Figure 2:**
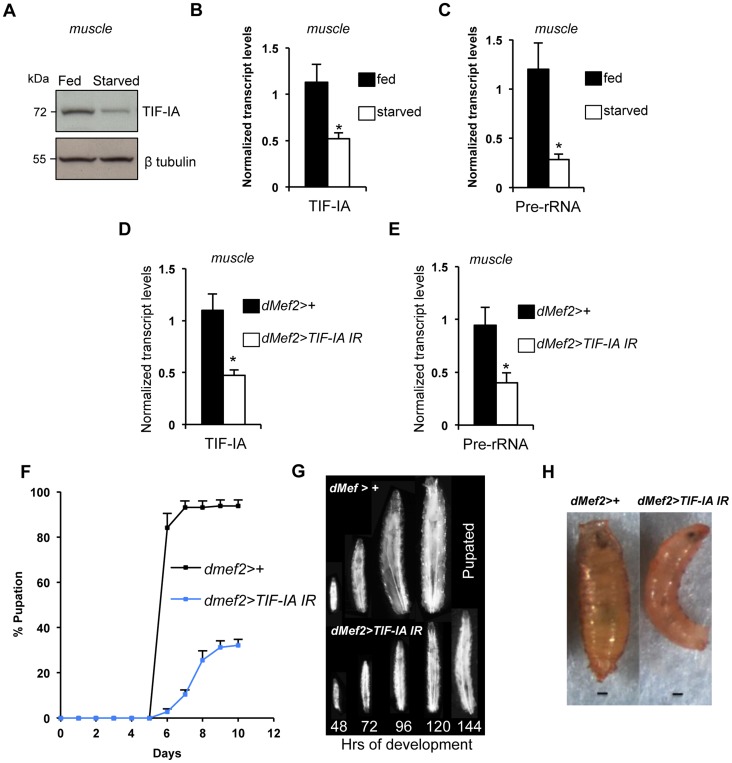
TIF-IA function is required in muscle to maintain overall body growth and development. (A) Immunoblot indicates TIF-IA protein levels were reduced in 24 hr starved muscle compared to fed muscle, at 72 hr AEL. β tubulin levels indicate loading control. (B) qPCR indicates *TIF-IA* mRNA levels were reduced in 24 hr starved muscle compared to fed muscle, at 72 hr AEL. Data normalized to *β tubulin*. (^*^ *P* = 0.009, Student's t-test). (C) qPCR indicates *pre-rRNA* levels were reduced in 24 hr starved muscle compared to fed muscle, at 72 hr AEL. Data normalized to *β tubulin*. (^*^ *P* = 0.0059, Student's t-test). (D) qPCR indicates *TIF-IA* mRNA levels were reduced in *dMef2>TIF-IA-IR* muscle compared to control larval muscle (*dMef2>+*), at 72 hr AEL. Data normalized to *β tubulin*. (^*^ *P* = 0.0022, Student's t-test). (E) qPCR indicates *pre-rRNA* levels were reduced in *dMef2>TIF-IA-IR* muscle compared to *dMef2>+* (control) larval muscle, at 72 hr AEL. Data normalized to *β tubulin*. (^*^ *P* = 0.015, Student's t-test). (F) Developmental timing from larval hatching to pupation of *dMef2>+* and *dMef2>TIF-IA IR* animals, n = 134, n - number of larvae assessed per genotype, (mean time to pupation: *dMef2>+*, 6.1 days and *dMef2>TIF-IA*, 7.8 days, ^*^ *P*<0.05, Mann-Whitney U test). (G) Representative images of *dMef2>+* (top) and *dMef2>TIF-IA IR* (bottom) larvae. Numbers at the bottom of the panel indicates hours AEL. (H) Representative images of *dMef2>+* and *dMef2>TIF-IA IR* pupae, scale bar-200 µm. All error bars indicate SEM.

As well as controlling cell-autonomous growth, TOR activity in specific larval tissues is required for overall body growth in *Drosophila*. For example, reduced TOR signaling in larval muscle [28] and fat [25], [26] leads to reduced body growth. Given the importance of ribosome synthesis as an effector of TOR in the control of cell-autonomous growth, we examined whether TIF-IA-dependent ribosome synthesis could also exert non-autonomous effects on body growth. We first examined larval muscle. As with whole larvae, we found that protein starvation decreased both TIF-IA protein (Figure 2A) and mRNA (Figure 2B), and also pre-rRNA (Figure 2C) in larval muscle. To explore the consequence of this reduction in TIF-IA levels, we examined the effects of RNAi-mediated knockdown of TIF-IA in muscle, using a *UAS-TIF-IA* inverted repeat (IR) line. Ubiquitous expression of this *TIF-IA IR* line in larvae using the *daughterless (da)-GAL4* driver (*da>TIFIA-IR*) phenocopied *tif-ia* mutants, and led to reduced TIF-IA protein levels (Figure S1B) and larval growth arrest (Figure S1A). Both of these effects were fully reversed by co-expression of a *UAS-TIF-IA* transgene (Figure S1A), confirming the specificity of the *UAS-TIF-IA IR* line. We then used the *UAS-TIF-IA IR* line to knock down TIF-IA specifically in muscle (using the *dMef2-GAL4* driver – Figure S2). We found that RNAi-mediated knockdown of TIF-IA muscle mimicked the decrease in both *TIF-IA* mRNA (Figure 2D) and pre-rRNA (Figure 2E) levels following starvation. When we monitored larval growth and development, we observed that *dMef2>TIF-IA IR* larvae were smaller than age-matched control larvae (Figure 2G). Moreover, *dMef2>TIF-IA IR* larvae were significantly delayed in pupal development with respect to control (*dMef2>+*) larvae (Figure 2F), and only approximately 20% of *dMef2>TIF-IA IR* larvae formed pupae. These *dMef2>TIF-IA IR* pupae were malformed compared to control (*dMef2>+*) pupae (Figure 2H). We examined feeding by transferring *dMef2>+* (control) and *dMef2>TIF-IA IR* larvae onto yeast paste colored with blue food dye. After 4 hours, we observed that both the control and *TIF-IA IR* larvae contained blue food in their guts (Figure S3), suggesting that knockdown of TIF-IA in larval muscle did not impair feeding. Together, these findings indicated that TIF-IA-dependent ribosome synthesis in muscle is required to maintain normal body growth and development.

**Figure 3:**
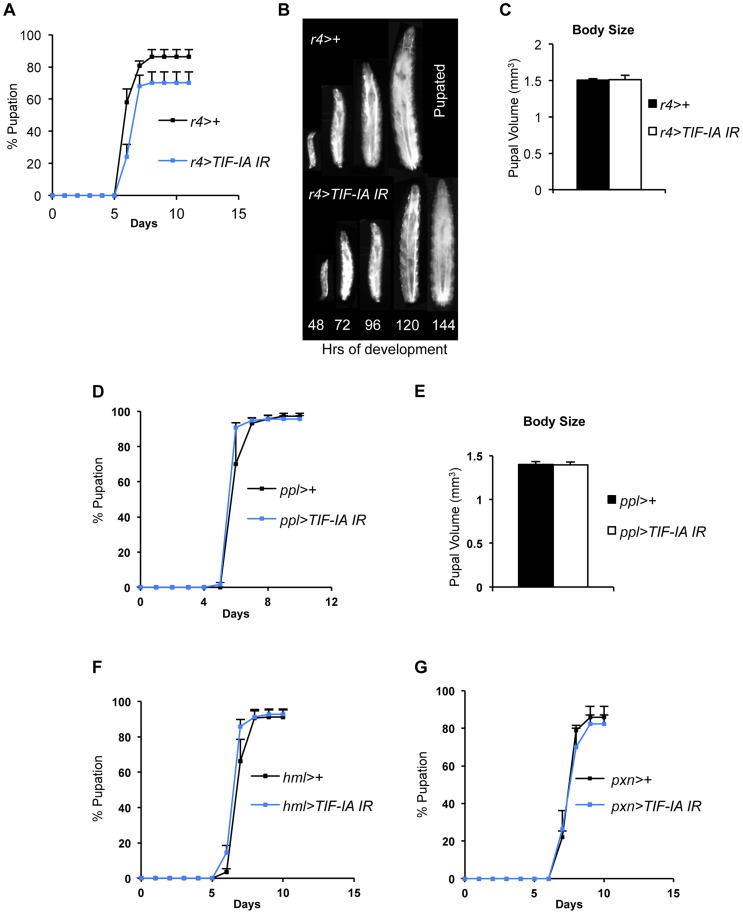
Fat-specific and lymph gland-specific TIF-IA inhibition has modest effects on growth and development. (A) Developmental timing from larval hatching to pupation of *r4>+* and *r4>TIF-IA IR* animals, n = 158, n - number of larvae assessed per genotype, (mean time to pupation: *r4>+*, 6.4 days vs. *r4>TIF-IA IR*, 6.7 days, ^*^ *P*<0.05, Mann-Whitney U test). (B) Representative images of *r4>+* (top) and *r4>TIF-IA IR* (bottom) larvae. Numbers at the bottom of the panel indicates hours AEL. (C) Pupal volume of *r4*>+ (n = 100) and *r4>TIF-IA IR* pupae (n = 28), n - number of pupae per genotype, (*P* = 0.92, Student's t-test). (D) Developmental timing from larval hatching to pupation of *ppl>+* and *ppl>TIF-IA IR* animals, n = 120, n - number of larvae assessed per genotype, (mean time to pupation: *ppl>+* 6.1 days vs. *ppl>TIF-IA IR* 6.1 days, not significant, Mann-Whitney U test). (E) Pupal volume of *ppl*>+ (n = 41) and *ppl>TIF-IA IR* pupae (n = 44), (*P* = 0.92, Student's t-test). (F) Developmental timing from larval hatching to pupation of *hml>+* and *hml>TIF-IA IR* animals, n = 192, n - number of larvae assessed per genotype, (mean time to pupation: *hml>+*, 7.2 days vs. *hml>TIF-IA IR*, 6.9 days, ^*^ *P*<0.05, Mann-Whitney U test). (G) Developmental timing from larval hatching to pupation of *pxn>+* and *pxn>TIF-IA IR* animals, n = 103, n - number of pupae counted per genotype, (mean time to pupation: *pxn>+*, 7.8 days vs. *pxn>TIF-IA IR*, 7.8 days, not significant, Mann-Whitney U test). All error bars indicate SEM.

We also examined the organismal effects of TIF-IA knockdown in other tissues. We used two fat body GAL4 drivers (*r4-GAL4* and *ppl-GAL4*) to express *UAS-TIF-IA IR* during larval development. We found that *r4>TIF-IA IR* larvae showed a modest, although statistically significant delay in both developmental timing - time from larval hatching to pupation (Figure 3A) - and growth (Figure 3B), but showed no significant change in body size compared to control (*r4>+*) animals (Figure 3C). Co-overexpression of *UAS-Tsc1* and *UAS-Tsc2* - negative regulators of TORC1 – using *r4-GAL4* led to marked reduction in body growth, thus confirming the effectiveness of the driver (Figure S4). We also found that *ppl>TIF-IA IR* larvae showed no significant difference in developmental timing (Figure 3D) or final body size (Figure 3E) compared to controls (*ppl>+*). We also examined the effects of TIF-IA knockdown in the larval lymph gland and hemocytes using two different drivers, *hemolectin (hml)-GAL4* and *peroxidasin (pxn)-GAL4*. In both cases, we observed no statistically significant decrease in larval development (Figure 3F, G). In fact, larval development was modestly, although significantly, accelerated in *hml>TIF-IA IR* larvae.

### TIF-IA function is required for muscle-specific effects of TOR on body growth

**Figure 4:**
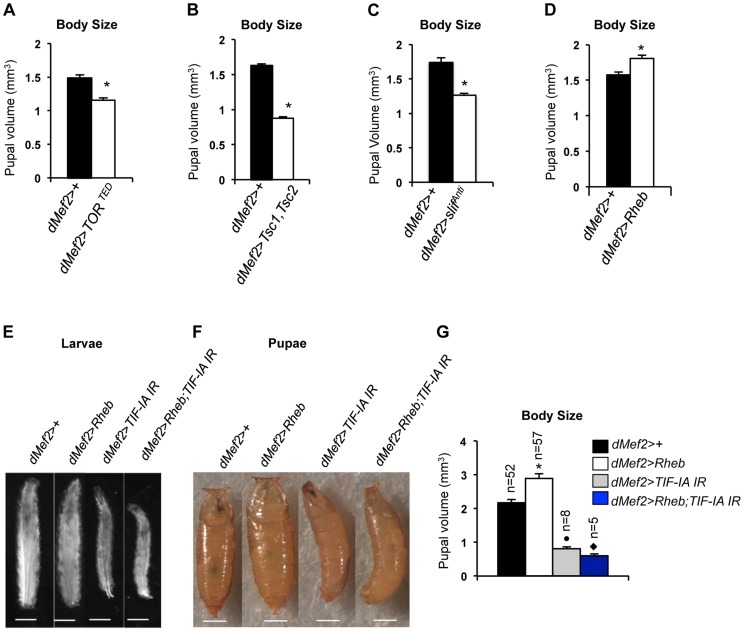
TOR activity in muscle is required and sufficient to promote body growth and TIF-IA inhibition in muscle blocks Rheb induced body growth. (A) Pupal volume of *dMef2*>+ and *dMef2>TOR^TED^* pupae, n = 29, n - number of pupae per genotype, (^*^ *P* = 2.47×10^−7^, Student's t-test). (B) Pupal volume of *dMef2*>+ and *dMef2>Tsc1,Tsc2* pupae, n>55, n - number of pupae per genotype, (^*^ *P* = 1.08×10^−51^, Student's t-test). (C) Pupal volume of *dMef2*>+ and *dMef2>slif^Anti^* pupae, n>80, n - number of pupae per genotype, (^*^ *P* = 5.14×10^−10^, Student's t-test). (D) Pupal volume of *dMef2*>+ and *dMef2>Rheb* pupae, n>38, n - number of pupae per genotype, (^*^ *P* = 0.003, Student's t-test). (E-F) Representative figures of larvae and pupae of indicated genotypes, scale bar-500 µm. (G) *dMef2>Rheb* animals showed increased pupal volume (White bar, ^*^ *P*<0.0001, One-way ANOVA and Tukey's post test) compared to *dMef2>+* control. Muscle specific inhibition of TIF-IA *(dMef2>TIF-IA IR)* reduced pupal volume, with respect to *dMef2>+* control (Grey bar, ^•^ *P* = 0.0003, One-way ANOVA and Tukey's post test). TIF-IA knockdown in muscle abrogated the Rheb-induced increase in pupal volume (Blue bar, ^⧫^ *P*<0.0001, One-way ANOVA and Tukey's post test), n - number of pupae per genotype. All error bars indicate SEM.

TOR activity in muscle is required for normal larval growth and development [28]. We confirmed this finding by inhibiting TOR in the muscle by two different methods, expression of a dominant negative form of TOR in muscle (*dMef2>TOR^TED^*) [32] and co-overexpression of *UAS-Tsc1* and *UAS-Tsc2* - negative regulators of TORC1 - in muscle (*dMef2>Tsc1,Tsc2*). We measured pupal volume, as an indicator of final body size. Our data showed that in both cases, inhibition of TOR in larval muscle reduced pupal volume (Figure 4A, B). Amino acid availability is an important activator of TOR kinase signaling, and we also found that knockdown of the amino acid transporter *slimfast* (using a *UAS-slif^Anti^* antisense [25]), in the larval muscle led to a significant reduction in pupal volume (Figure 4C). Finally, we also examined whether over-activation of TOR in muscle was sufficient to drive systemic growth. We found that overexpression of Ras homolog enriched in brain (Rheb), an upstream activator specifically of TORC1, in muscle (*dMef2>Rheb*) was sufficient to increase pupal volume compared to control (*dMef2>+*) pupae (Figure 4D). Together, these findings suggest that TOR activity in muscle is both necessary and sufficient to control overall systemic growth.

We next examined whether TIF-IA function was required for these muscle effects of TOR. As described above, overexpression of Rheb in muscle led to increased body size, as indicated by larger larvae (Figure 4E) and increased pupal volume (Figure 4F), while RNAi-mediated knockdown of TIF-IA showed the opposite effects. We found that co-expression of *UAS-TIF-IA IR* (*dMef2>Rheb;TIF-IA IR*) phenocopied *dMef2>TIF-IA IR* animals and abrogated the Rheb induced increase in body size. We quantified the pupal volume and found that reducing TIF-IA in muscle reduced the Rheb induced increase in pupal volume (Figure 4G). Overall, these data indicated that TIF-IA activity in muscle is required for TOR signaling to drive systemic growth.

### TIF-IA function in muscle is required to maintain systemic insulin signaling

**Figure 5:**
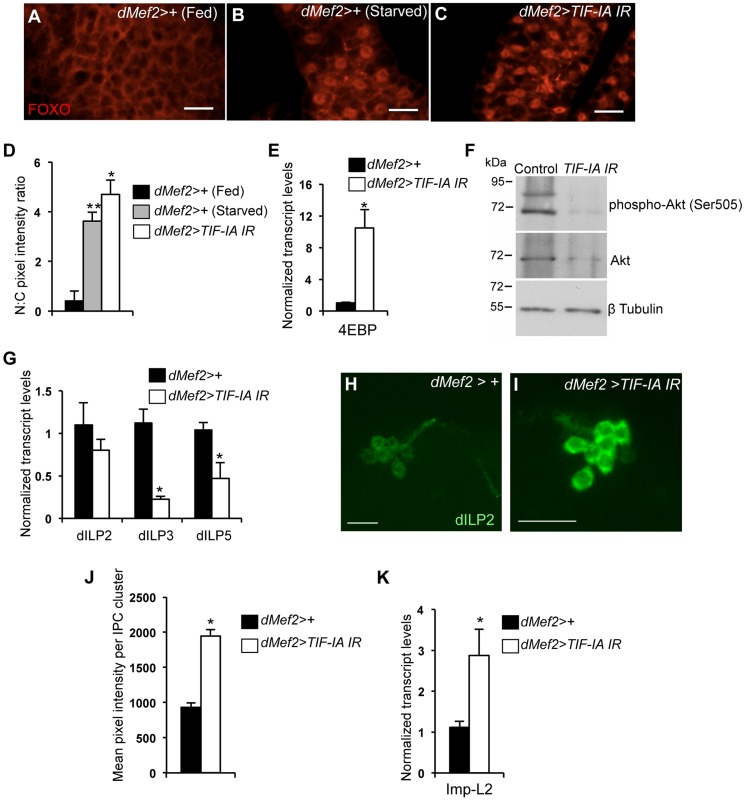
Muscle-specific TIF-IA inhibition reduces systemic insulin signaling. (A–C) Representative fat body images indicating FOXO (red) subcellular localization in (A) *dMef2>+* (Fed), (B) *dMef2>+* (Starved) and (C) *dMef2>TIF-IA IR* larvae, scale bar-500 µm. (D) Quantification indicating mean (N∶C, Nuclear∶Cytoplasmic) ratio of pixel intensity per fat body cell of *dMef2>+* (Starved) (Grey bar, ^**^ *P*<0.001, One-way ANOVA and Tukey's post test) and *dMef2>TIF-IA IR* (White bar, ^*^ *P*<0.001, One-way ANOVA and Tukey's post test) animals, compared to fed control (*dMef2>+*) animals. 21 cells/genotype were scored. (E) qPCR indicates *4EBP* mRNA levels were increased in *dMef2>TIF-IA IR* larvae compared to *dMef2>+* control (^*^ *P* = 0.002, Student's t-test). Data normalized to *β tubulin* mRNA. (F) Immunoblots indicate phospho Akt (Ser505), Akt and βtubulin levels in control (*dMef2>+*) and TIF-IA IR (*dMef2>TIF-IA IR*) larvae. (G) *dMef2>TIF-IR* larvae had reduced *dILP3* mRNA (^*^ *P* = 0.0003, Student's t-test) and *dILP5* mRNA (^*^ *P* = 0.015, Student's t-test) levels but *dILP2* mRNA (*P* = 0.14, Student's t-test) levels were unaltered, compared to *dMef2>+* control. Data normalized to *β tubulin* mRNA. (H–I) Representative images of larval brain insulin producing cells (IPC) at 96 hr AEL, indicating dILP2 protein accumulation of (H) *dMef2>+* and (I) *dMef2>TIF-IA IR* animals, scale bar-20 µm. (J) Quantification showing mean pixel intensity/IPC cluster of *dMef2>+* (n = 16) and *dMef2>TIF-IA IR* (n = 16) animals, n – number of IPC cluster assessed per genotype, images quantified with Image J software, (^*^ *P* = 1.21×10^−10^, Student's t-test). (K) qPCR indicates *Imp-L2* mRNA levels were induced in *dMef2>TIF-IA IR* larval muscle compared to *dMef2>+* (control), (^*^ *P* = 0.025, Student's t-test). Data normalized to *β tubulin* mRNA. All error bars indicate SEM.

The insulin pathway is the major endocrine regulator of body growth in larvae. Under nutrient-rich conditions, several dILPs are expressed and released into the larval hemolymph [33]. These dILPs then bind to a single insulin receptor in target cells and promote growth [26]. In contrast, starvation leads to reduced systemic insulin signaling and decreased growth. We therefore explored whether the growth inhibitory effects of muscle-specific TIF-IA knockdown were due to reduced systemic insulin signaling. Under nutrient rich conditions, high level of insulin signaling leads to activation of Akt kinase and phosphorylation and nuclear exclusion of the FOXO transcription factor. But when insulin signaling is reduced, FOXO relocalizes to the nucleus and activates target genes such as eIF4E-Binding Protein (4EBP). Therefore, changes in FOXO nuclear localization and transcriptional activity serve as a reliable ‘read-out’ of insulin signaling [34]–[36]. As previously reported, we found that FOXO was excluded from nuclei in fat body cells from fed larvae (Figure 5A), but showed strong nuclear accumulation in fat body cells from starved larvae (Figure 5B). When we knocked-down TIF-IA levels in muscle (*dMef2>TIF-IA IR*), FOXO showed strong, statistically significant nuclear accumulation in fat body cells (Figure 5C, D). We next measured the levels of 4EBP, a FOXO target gene, and found that *dMef2>TIF-IA IR* larvae had increased 4EBP mRNA levels with respect to control (*dMef2>+*) larvae (Figure 5E). Finally we measured the examined levels of phosphorylated Akt – the kinase downstream of insulin signaling that is responsible for phosphorylation and inhibition of FOXO. Using western blotting with an anti-phospho Akt (Ser505) antibody, we found that *dMef2>TIF-IA IR* had markedly reduced levels of phospho Akt compared to control (*dMef2>+*) larvae (Figure 5F). Levels of total Akt were also lower, but much less so than the suppression in levels of phosphorylated Akt. Together these data suggest that TIF-IA knockdown in muscle leads to reduction in systemic insulin signaling.

An important source of dILPs is a cluster of neurosecretory cells in the larval brain [33], [37]. These cells secrete three dILPs (2, 3 and 5), and expression and/or release of these dILPs are suppressed upon protein starvation [26]. Moreover, loss of these neurons leads to slow growing and small larvae [33], [37], [38]. We found that *dMef2>TIF-IA IR* larvae had reduced dILP3 and dILP5 mRNA levels compared to control (*dMef2>+*) larvae, while dILP2 mRNA levels were unaltered (Figure 5G). Previous studies showed that nutrient-deprivation leads to reduced dILP2 secretion and hence increased retention in the neurosecretory cells [26]. This retention can be easily visualized by staining with anti dILP2 antibodies. Using, this approach we observed an increase in dILP2 staining in the neurons of *dMef2>TIF-IA IR* larvae compared to control larvae (Figure 5H, I, J). Together, these data suggest that one mechanism by which reduced TIF-IA activity in muscle suppresses peripheral insulin signaling is by reduced expression and release of brain-derived dILPs.

In addition to the dILPs, other secreted factors can influence insulin signaling in *Drosophila*. One factor is Imaginal morphogenesis protein-L2 (Imp-L2), which is the *Drosophila* homolog of insulin-like growth factor binding protein 7 (IGFBP7) [39]. Imp-L2 can bind to dILPs and inhibit their ability to signal through the insulin receptor [39]–[41]. Moreover, a recent report showed that mitochondrial perturbation in adult muscle leads to increased Imp-L2 expression and subsequent suppression of systemic insulin signaling [42], [43]. We found that *dMef2>TIF-IA IR* larvae had upregulated *Imp-L2* mRNA levels in their muscle compared to control (*dMef2>+*) larvae (Figure 5K). These data suggest that upregulation of Imp-L2 may provide another mechanism by which perturbation of TIF-IA in muscle suppresses systemic insulin signaling. Indeed, we found that overexpression of Imp-L2 in the muscle led to delayed larval development and reduced pupal size (Figure S5).

**Figure 6:**
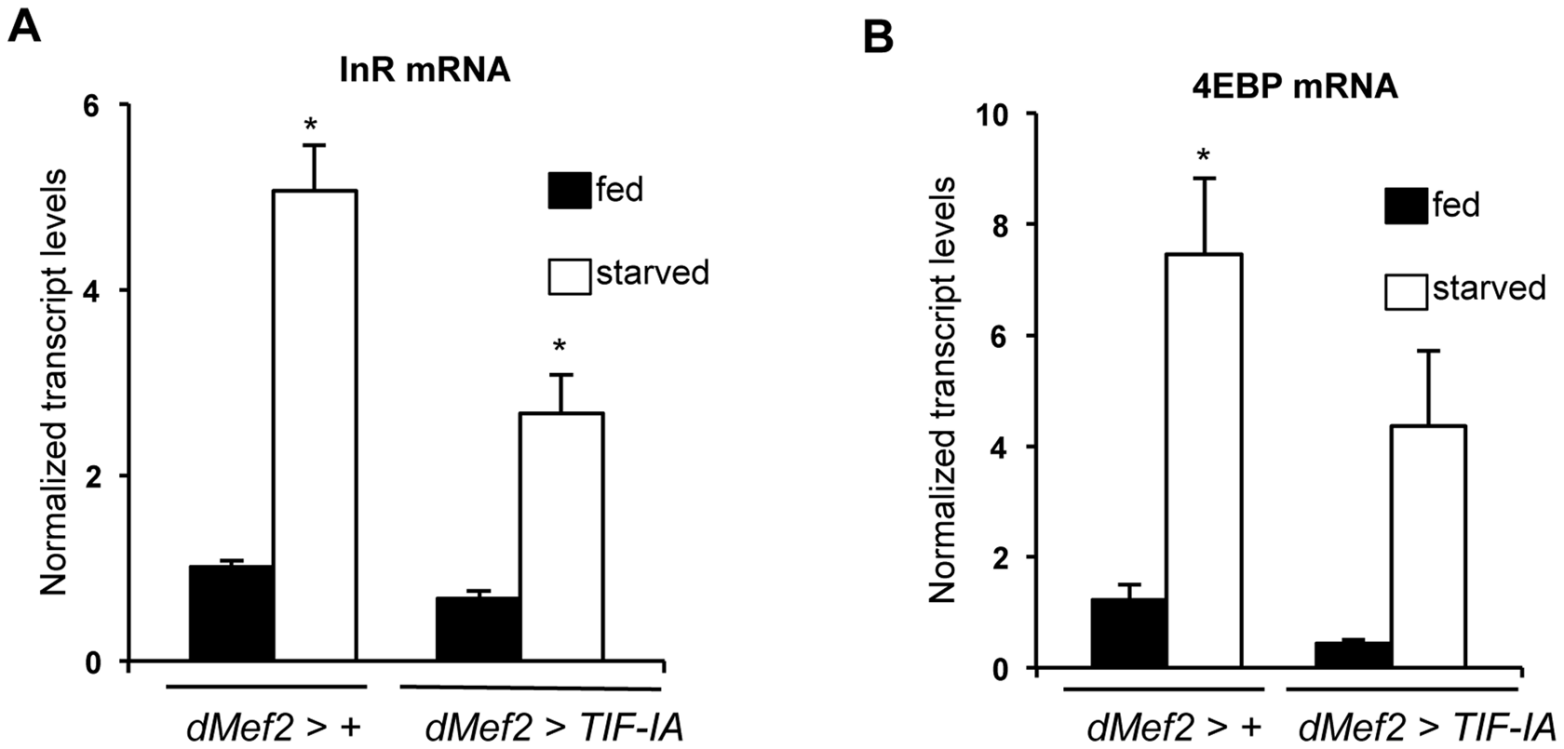
TIF-IA overexpression in muscle can partially reverse the effects of starvation on FOXO-dependent genes. (A) Data present mean +/− SEM values from qPCR analysis of *InR* mRNA levels in fed and starved larvae of *dMef2>+* and *dMef2>TIF-IA* animals. Starvation increased *InR* mRNA levels, compared to fed controls (^*^ *P*<0.0001, One-way ANOVA and Tukey's post test). Overexpression of TIF-IA in muscle significantly suppressed this starvation-mediated InR induction (^*^ *P*<0.0001, One-way ANOVA and Tukey's post test). Data normalized to *β tubulin* mRNA. (B) Data present mean +/− SEM values from qPCR analysis of *4EBP* mRNA levels in fed and starved larvae of *dMef2>+* and *dMef2>TIF-IA* animals. Starvation increased *4EBP* mRNA levels, compared to fed controls (^*^ *P* = 0.0003, One-way ANOVA and Tukey's post test). Overexpression of TIF-IA in partially suppressed the starvation mediated 4EBP mRNA induction, although not to a statistically significant level (*P* = 0.125, One-way ANOVA and Tukey's post test). Data normalized to *β tubulin* mRNA. All error bars indicate SEM.

Our data suggest that TIF-IA function in muscle is required to maintain systemic insulin signaling in fed animals. We next examined whether TIF-IA-mediated ribosome synthesis in muscle may provide one mechanism to couple nutrient availability to systemic insulin signaling. We overexpressed a *UAS-TIF-IA* transgene in muscle (*dMef2>TIF-IA*) and observed a very slight, but statistically significant acceleration in development compared to (*dMef2>+*) larvae (Figure S6A), although final body size was not affected (Figure S6B). Similar effects were observed with a second, independent *UAS-TIF-IA* transgene (Figure S6C, S6D). We then examined effects of muscle TIF-IA overexpression in starved animals. When larvae are deprived of dietary protein, insulin signaling is reduced leading to upregulated levels of FOXO target genes such as *4EBP* and *InR*, an effect we observed here following 24 hr starvation. However, when we overexpressed TIF-IA in muscle (*dMef2>TIF-IA*), the starvation-induced increase in both *4EBP* and *InR* mRNA was partially reversed compared to control (*dMef2>+*) larvae (Figure 6A, B). This result suggests that TIF-IA function in muscle can, in part, couple nutrient availability to systemic insulin signaling.

### Reduced FOXO levels or knockdown of Imp-L2 partially reverses the growth defects caused by muscle TIF-IA inhibition

**Figure 7:**
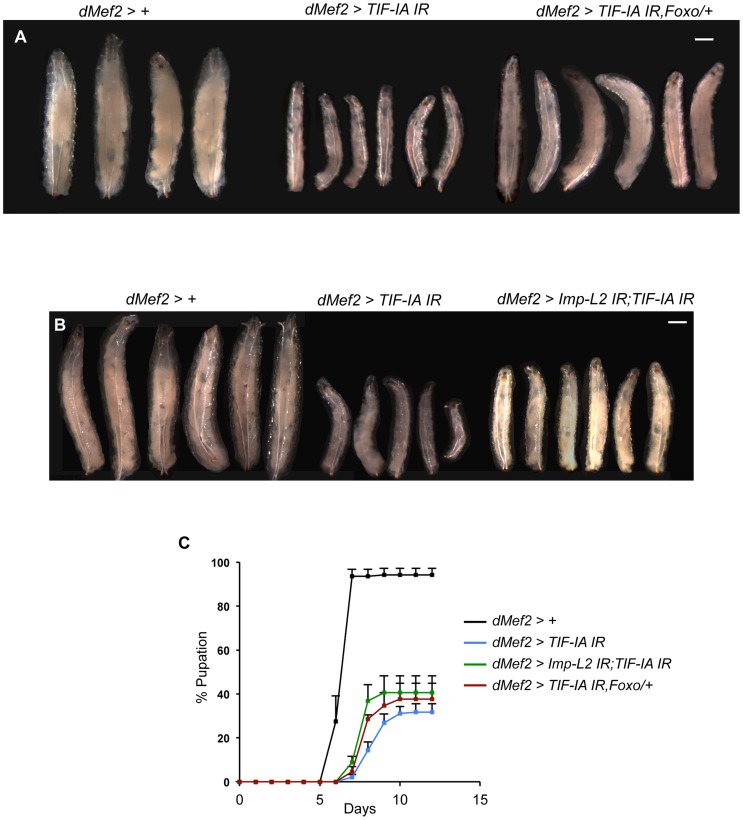
Reduction of Imp-L2 levels or removal of one copy of *foxo* (*foxo^25^*/+) partially rescues *dMef2>TIF-IA IR* induced body growth defect and developmental delay. (A–B) Representative images of larvae of indicated genotypes. The images were captured when control larvae (*dMef2>+*) reached wandering third instar stage. The larval body areas were measured and analyzed: A) *dMef2>TIF-IA IR* larvae were 33.9% (+/−1) of control (*dMef2>+*) larvae size. *dMef2>TIF-IA IR*, *foxo/+* were 57.8% (+/−3) of control larvae size (P<0.01 vs *dMef2>TIF-IA IR* larvae). B) *dMef2>TIF-IA IR* larvae were 31.4% (+/−4.6) of control (*dMef2>+*) larvae size. *dMef2>TIF-IA IR*, *Imp-L2 IR* were 45.7% (+/−1.5) of control larvae size (P<0.05 vs *dMef2>TIF-IA IR* larvae). Scale bar-500 µm (C) Developmental timing of larvae of indicated genotypes from hatching to pupation. Mean time to pupation for each genotype: *dMef2>+*, 6.7 days; *dMef2>TIF-IA IR*, 8.7 days; *dMef2>TIF-IA IR*, *foxo^25^/+*, 8.1 days (^*^ *P* = 0.05 vs. *dMef2>TIF-IA IR* larvae, Mann-Whitney U test); *dMef2>TIF-IA IR*, *Imp-L2 IR*, 7.9 days (^*^ *P* = 0.05 vs. *dMef2>TIF-IA IR* larvae, Mann-Whitney U test).

The findings presented here suggest that TIF-IA function in muscle is required for normal nutrient-dependent systemic insulin signaling and growth. Hence, upon knockdown of TIF-IA in muscle, we saw reduced growth and delayed development. To further implicate a role for reduced insulin signaling in these effects, we tested whether restoring insulin signaling to some degree could have any effect on the phenotypes we observed. To achieve this we examined partial loss of negative regulators of insulin signaling. We first tested the effects of *reducing foxo* gene dosage. We found that the decrease in larval growth seen in *dMef2>TIF-IA IR* larvae was partially reversed in larvae that were heterozygous for a loss-of-function mutation in *foxo (foxo^25^)* (Figure 7A). We next examined the effects of reducing the levels of Imp-L2, whose expression was increased in *dMef2>TIF-IA IR* larval muscle. We found that co-expression of a *UAS-Imp-L2* inverted repeat (IR) line with the *UAS-TIF-IA IR* in muscle, also partially reversed the growth defects seen with expression of *UAS-TIF-IA IR* alone (Figure 7B). Loss of one copy of *foxo (foxo^25^/+)* alone or expression of *UAS-Imp-L2 IR* alone in the muscle had no effects on larval size (Figure S7). When we measured developmental timing, we also saw that both the delayed larval development and reduced numbers of pupating larvae seen in *dMef2>TIF-IA IR* larvae were partially reversed in larvae that either were heterozygous for *foxo^25^*, or which co-expressed *UAS-Imp-L2 IR* in the muscle (Figure 7C). These experiments provide genetic evidence that muscle TIF-IA function is required for normal larval growth and development at least in part by maintaining systemic insulin signaling.

## Discussion

The major finding of our work is that under nutrient-rich conditions TIF-IA-dependent regulation of muscle ribosome synthesis is required to maintain systemic insulin signaling and body growth.

Work in yeast, mammalian cell culture and *Drosophila* indicates that TIF-IA links nutrient availability and TOR signaling to rRNA synthesis [4]–[10]. Here we show that in growing tissues *in vivo* one mechanism by which nutrient/TOR signaling functions is through maintaining TIF-IA levels. Recent studies in yeast also showed TIF-IA levels were reduced following pharmacological inhibition of TOR [17]. Moreover, in previous work, we showed that maintaining high levels of TIF-IA expression could reverse the decrease in rRNA synthesis caused by amino acid starvation in *Drosophila* larvae [6]. Hence, control of TIF-IA levels represents one mechanism by which nutrient availability and TOR signaling can control the synthesis of rRNA. TOR has also been reported to indirectly control site-specific phosphorylation of TIF-IA, and this phosphorylation modulates TIF-IA nucleolar localization [9]. Hence, TOR may impact TIF-IA function in several ways.

When we mimicked the starvation induced decrease in muscle TIF-IA mRNA levels by RNAi-mediated knockdown, we observed that larvae were slower growing and failed to develop. This phenotype was not simply due to a gross motor defect, since the larvae were able to crawl normally and ingest food. Studies from Demontis and Perrimon [28] describe a similar reduced growth phenotype following inhibition of TOR signaling in larval muscle. Here, we extended this work to show that increased TOR in muscle leads to a larger overall body size, and that this effect required intact TIF-IA function.

Our data implicate changes in insulin signaling as underlying the effects of TIF-IA-dependent ribosome synthesis in muscle on overall body growth and development. Our findings also suggest that the ability of dietary nutrients to stimulate and maintain systemic insulin rely, in part, on maintaining TIF-IA levels and function in muscle. Muscle TIF-IA appeared to control insulin signaling by at least two mechanisms. First, we saw that expression of brain-derived dILPs required normal muscle TIF-IA function. The expression and release of dILPs (2,3 and 5) from a cluster of neurosecretory cells [33], [37] in the brain is regulated by signals from other tissues. Hence, the changes in systemic insulin signaling that we saw following inhibition of TIF-IA in muscle could be explained by a role for muscle-derived secreted factors (often termed myokines). In mammals, muscle has been shown to secrete many factors, including a host of cytokines, and secretion of these factors is often controlled by nutrients [44]–[50]. In *Drosophila*, the full complement of factors secreted from muscle is not clear [49]. Nevertheless, one or more secreted factors could potentially signal to the brain to promote dILP release. Indeed, a recent study showed that suppression of ribosome synthesis by overexpression of Mnt in adult muscle led to release of myoglianin, a myostatin-like myokine, which induced remote effects on fat body function [51]. Also, activin signaling in adult muscle can remotely control dILP release and systemic insulin signaling [52]. Second, we saw that knockdown of TIF-IA in muscle led to an increase in expression of Imp-L2, a secreted protein that functions to suppress insulin signaling [39]. A recent paper showed that perturbation of mitochondrial function in *Drosophila* muscle can also lead to upregulation of Imp-L2 expression [42]. Together with our data, this finding suggests that upregulation of Imp-L2 may be a common response triggered by metabolic stress in muscle cells. Importantly, we were able to partially rescue both the reduced growth and delayed development seen with muscle knockdown of TIIF-IA by either loss of one copy of *foxo* or RNAi-mediated knockdown of muscle Imp-L2. In both cases, the rescue was partial probably because neither genetic manipulation would be predicted to completely restore systemic insulin signaling. Nevertheless, the findings provide further support for our model that muscle-specific ribosome synthesis can control systemic insulin signaling and body growth.

A previous report described how inhibition of PI3K/TOR signaling in muscle led to both reduced muscle cell size and a non-autonomous reduction in size of other tissues and overall body size [28]. These non-autonomous effects were proposed to be mediated through altered endocrine signaling from the muscle to other tissues, although it is unclear whether this occurred solely as a result of reduced muscle cell size, or whether it reflects a cell size-independent role for TOR in controlling the endocrine function of muscle. Our findings here suggest that altered insulin signaling is one important endocrine response that links changes in muscle ribosome synthesis to altered physiology and growth in other tissues, although as with the effects of TOR we cannot discern whether this occurs only due to reduced muscle cell size. Interestingly we showed that inhibition of TIF-IA in the fat body had only a weak non-autonomous effect on body growth, although TIF-IA knockdown can limit fat cell size and ploidy [6]. Thus the mechanisms that couple TIF-IA and ribosome synthesis in muscle to the endocrine control of systemic insulin may not operate in the fat body. Ultimately, it is likely that the role for TIF-IA and ribosome synthesis in controlling overall body growth depends on a combination of cell-autonomous and non-autonomous influences. For example, inhibition of ribosome synthesis in the prothoracic gland was shown to extend larval development by altering endocrine ecdysone hormone signaling [53].

Muscle is a metabolically active tissue that probably has a high demand for continued ribosome biogenesis and protein synthesis to maintain autonomous growth. Our studies suggest that muscle ribosome synthesis may also act as a checkpoint for overall body growth. If muscle ribosome synthesis is perturbed (e.g. by nutrient deprivation), this may cause muscle cells to trigger a suppression of systemic insulin signaling to limit body growth. In using ribosome synthesis as a checkpoint for controlling systemic insulin, muscle cells may simply sense and respond to general changes in bulk translation. Alternatively, altered translation of a select subset of mRNAs may influence either Imp-L2 expression or the ability of muscle to remotely control brain dILP expression. In either case, our findings suggest that larval muscle is also an important nutrient-sensing tissue, in addition to the fat body, that can control systemic insulin signaling via endocrine signaling. The endocrine mechanisms by which either fat or muscle control systemic insulin signaling are nor clear and may be different in both cases. However, it seems that both tissues rely on protein synthesis, although perhaps through different mechanisms. Our data suggest that control of rRNA synthesis is an important limiting step in muscle, while previous work suggests that regulation of tRNA synthesis and signaling via Myc is important in fat [36], [54], [55].

The ‘checkpoint’ response to perturbation of muscle ribosome synthesis may be important for controlling not just growth, but also other organismal responses. For example, upon starvation or other environmental stressors, a reduction of muscle TIF-IA and ribosome synthesis may function to suppress systemic insulin signaling to alter whole body metabolism in order to maintain animal survival under adverse conditions. Reducing insulin signaling has been well described as mediator of stress resistance and extended lifespan in many animals including *Drosophila*, *C. elegans* and mice [56]–[58]. Indeed, a recent report showed that elevated Imp-L2 in *Drosophila* muscle increased adult lifespan [42], [43]. Also, overexpression of 4E-BP, a translational repressor, in muscle [59] or in whole organism [60] leads to stress resistance, and extended lifespan. Thus control of muscle protein synthesis, possibly by regulating ribosome biogenesis, may be a common mechanism to control stress responses and lifespan by regulating whole-body insulin signaling.

## Materials and Methods

### Fly strains and husbandry

All stocks and crosses were raised at 25°C and maintained on a media containing 100 g *Drosophila* Type II agar, 1200 g cornmeal, 770 g Torula yeast, 450 g sugar, 1240 g D-glucose, 160 ml acid mixture of propionic acid and phosphoric acid per 20 L of water. The following fly stocks were used: *w^1118^*; *yw*; *UAS-TIF-IA*; *UAS-TIF-IA IR* (v20334, Vienna *Drosophila* RNAi Center, VDRC); *UAS-Tsc1*, *UAS-Tsc2*; *tor^ΔP^*/*CyO*; *s6k^L1^*/*TM6B*; *UAS-Rheb*; *UAS-slif^Anti^; UAS-TOR^TED^*; *UAS-GFP; UAS-Imp-L2 IR* (15009-R3, NIG, Japan); *foxo^25^/TM6B*, *dMef2-GAL4*; *da-GAL4; r4-GAL4; ppl-GAL4; hml-GAL4; pxn-GAL4*. For all GAL4/UAS experiments, homozygous GAL4 lines were crossed to the relevant UAS line(s) and the larval progeny were analyzed. Control animals were obtained by crossing the relevant homozygous GAL4 line to either *w^1118^; +; +* or *yw; +; +*, depending on the genetic background of the particular experimental UAS transgene line.

### Egg collection

Adult flies were allowed to lay eggs on grape juice agar plates supplemented with yeast paste for 4 hours (hr) at 25°C. 24 hr after egg laying (AEL) all hatched larvae were transferred to food vials with a thin brush, in groups of 45–50 larvae/vial and allowed to develop.

### Larval starvation

For all experiments, whole larvae were starved by floating on sterile 20% sucrose in 1× Phosphate Buffered Saline (PBS) at 72 hr AEL for indicated times. Subsequently, larvae were collected and processed as per experimental requirements. Fed larvae were collected at 72 hr AEL.

### Preparation of protein extracts, immunoblotting and antibodies

Whole larval or larval muscle tissue extracts were prepared by lysing 72 hr AEL larvae in 4× protein sample buffer (240 mM Tris-HCl pH 6.8, 8% SDS, 5%β-mercaptoethanol, 40% glycerol, 0.04% bromophenol blue) with a motorized pestle, boiling for 4 minutes at 95°C and immediately loading the samples onto a SDS-PAGE gel. Immunoblotting was performed as previously described [54]. Antibodies used were βtubulin (E7, *Drosophila* Studies Hybridoma Bank), phospho-*Drosophila* Akt Ser505 (Cell Signaling Technology, 4054) and Akt (Cell Signaling Technology, 9272). Affinity-purified antibodies were generated against TIF-IA was raised by immunizing rabbits using the synthetic peptide CIVDKRPKNFDLSKSQEFDKQ, corresponding to residues 585–604 (Anaspec Inc.).

### Quantitative Real Time - PCR (qPCR)

Whole larval or larval muscle tissues were isolated at definite time points AEL (as indicated in the figure legends). Total RNA was extracted using TRIzol according to the manufacturer's instructions (Invitrogen; 15596-018). RNA samples were DNase treated as per manufacturer's protocol (Ambion; 2238G). The DNase treated RNA was reverse transcribed by Superscript II to make cDNA. This cDNA was used as a template to perform qRT-PCR reactions (BioRad Laboratories, MyIQ PCR machine using SYBR Green PCR mix) using specific primer pairs (sequences available upon request). Pre-rRNA levels were measured by using primer pairs against the internal transcribed spacer (ITS) region of 45S pre-rRNA transcript. qPCR data were normalized to *β tubulin* mRNA, whose levels we found were essentially unchanged across all the experimental conditions. The exception was the qPCR analyses of *tor* mutants, where values were corrected for *actin* mRNA levels. For each experiment, a minimum of 3 groups of 5–8 larvae was collected. Each experiment was independently repeated a minimum of 3 times.

### Pupation rates

Larvae were collected at 24 hr AEL and placed in food vials in equal numbers per vial (with a maximum density of 50 larvae per vial). The number of pupae in vials was counted every 24 hr. For each genotype, minimum of 3 replicates were used to calculate the mean percentage of pupae per timepoint.

### Pupal volume

Pupal volume was calculated as previously described [55].

### Microscopy

Larval and pupal images were obtained using a Zeiss Stereo Discovery V8 microscope using Axiovision software. Microscopy and image capture was performed at room temperature and captured images were processed using Photoshop CS5 (Adobe). For each experiment all larval and pupal images were captured using identical magnifications. Final figures were generated from these by cropping individual larvae and then simply rotating images to orient them in the same direction, without altering size or scale. These images were then assembled on a single black canvas in Photoshop. Larval sizes were assessed by using Photoshop to measure larval body areas from these microscope images. Tissue images and Differential Interference Contrast (DIC) images were captured by taking serial Z-stacks using the same magnification and time of exposure.

### Immunohistochemistry

Larvae were inverted using fine forceps in 1× PBS at particular time points (as indicated in the figure legends). Inverted larvae were fixed in 8% paraformaldehyde for 40 minutes, washed in 1× PBS-0.1% TritonX (PBST), blocked for 2 hr at room temperature in 1× PBST with 5% fetal bovine serum (FBS). Larvae were incubated overnight with primary antibody at 4°C, washed several times with 1× PBST and incubated with secondary antibody (1∶4000) for 2 hours, at room temperature. After few washes, fat bodies were isolated from these larvae using fine forceps and mounted on glass slides with cover slips using Vectashield (Vector Laboratories Inc., CA) mounting media. Primary antibodies used were rabbit anti-FOXO (from Marc Tatar) and rabbit anti-dILP2. Alexa Fluor 488 and 568 (Invitrogen) were used as secondary antibodies. Hoechst 33342 (Invitrogen) was used to stain nuclei. dILP2 immunostaining of larval brains was performed as described [54].

### Statistics

For all experiments, error bars represent standard error of mean (SEM). *P* values were computed by Student's t-test, using Microsoft Excel or Analysis of Variance (ANOVA) followed by Tukey's post-hoc test, using GraphPad prism (version 6). For developmental timing experiments, mean time to pupation was computed using Mann-Whitney U test using GraphPad prism (version 6). *P<0.05* was considered to be statistically significant, as indicated by asterisk (^*^) or as indicated in the figure legend.

## Supporting Information

Figure S1 **Co-expression of** ***UAS-TIF-IA*** **using** ***da-GAL4*** **driver rescued the growth defects in** ***da>TIF-IA IR*** **larvae.** (A) Representative images of 72 hr AEL larvae of indicated genotypes, scale bar-500 µm. (B) Immunoblot indicates TIF-IA protein levels in 72 hr AEL larvae of indicated genotypes. β tubulin levels indicate loading control.
(TIF)

Figure S2 ***dMef2-GAL4*** **drives expression in body wall muscle.** The *dMef2-GAL4* was used to drive expression of UAS-GFP. Wandering larvae were fixed, dissected and mounted. Representative images of A, A′) body wall muscle, B, B′) fat body, C, C′) gut and D, D′) salivary gland are shown. All images were captured using the same exposure and magnification.
(TIF)

Figure S3 **Muscle-specific TIF-IA inhibition does not affect larval food ingestion.** Representative images of *dMef2>+* and *dMef2>TIF-IA IR* larvae after 4 hrs of blue food (yeast paste colored with blue food dye) ingestion at 72 hr AEL, scale bar-500 µm.
(TIF)

Figure S4 **Overexpression of** ***UAS-Tsc1*** **and** ***UAS-Tsc2*** **in larval fat body inhibits body growth.** Representative image of *r4>+* and *r4>Tsc1,Tsc2* larvae when *r4>+* (control) larvae started wandering, scale bar-500 µm.
(TIF)

Figure S5 **Overexpression of Imp-L2 in muscle delays development and inhibits body growth.** (A) Developmental timing from larval hatching to pupation of *dMef2>+* (n = 183) and *dMef2>Imp-L2* (n = 180) animals, n - number of larvae assessed per genotype, mean time to pupation: *dMef2>+*, 6.79 days vs. *dMef2>Imp-L2*, 8.3 days, ^*^ *P* = 0.05, Mann-Whitney U test). (B) Pupal volume of *dMef2*>+ (n = 27) and *dMef2>Imp-L2* (n = 32) pupae, n - number of pupae per genotype, (^*^ *P* = 5.12×10^−11^, Student's t-test).
(TIF)

Figure S6 **Overexpression of TIF-IA in muscle modestly accelerates development but does not promote body growth.** (A) Developmental timing from larval hatching to pupation of *dMef2>+* and *dMef2>TIF-IA* animals, n = 138, n - number of larvae assessed per genotype, (mean time to pupation: *dMef2>+*, 6.9 days vs. *dMef2>TIF-IA*, 6.6 days, ^*^ *P* = 0.05, Mann-Whitney U test). (B) Pupal volume of *dMef2*>+ (n = 101) and *dMef2>TIF-IA* (n = 102) pupae, n - number of pupae per genotype, (*P* = 0.88, Student's t-test). (C–D) Experiments were performed using a second *UAS-TIF-IA* transgene. (C) Developmental timing from larval hatching to pupation of *dMef2>+* (n = 187) and *dMef2>TIF-IA* (n = 186) animals, n - number of larvae assessed per genotype, (mean time to pupation: *dMef2>+*, 7.1 days vs. *dMef2>TIF-IA*, 6.8 days, ^*^ *P* = 0.05, Mann-Whitney U test). (D) Pupal volume of *dMef2*>+ (n = 68) and *dMef2>TIF-IA* (n = 61) pupae, n - number of pupae per genotype, (*P* = 0.88, Student's t-test).
(TIF)

Figure S7 **Loss of one copy of** ***foxo*** **or knockdown of Imp-L2 in muscle does not affect larval size.** Representative images are shown of *dMef2-GAL4/+* (left), *dMef2/foxo^25^* (middle) and *UAS-Imp-L2 IR/+; dMef2-GAL4/+* (right) larvae. All images were taken when the control (*dMef2-GAL4/+*) larvae were at the wandering L3 stage. Scale bar-500 µm.
(TIF)
