# Supplementary figures and images for: TIF-IA-Dependent Regulation of Ribosome Synthesis in Drosophila Muscle Is Required to Maintain Systemic Insulin Signaling and Larval Growth
Source: PLoS Genet. 2014 Oct 30;10(10):e1004750. doi: 10.1371/journal.pgen.1004750 (PMC4214618; doi:10.1371/journal.pgen.1004750)

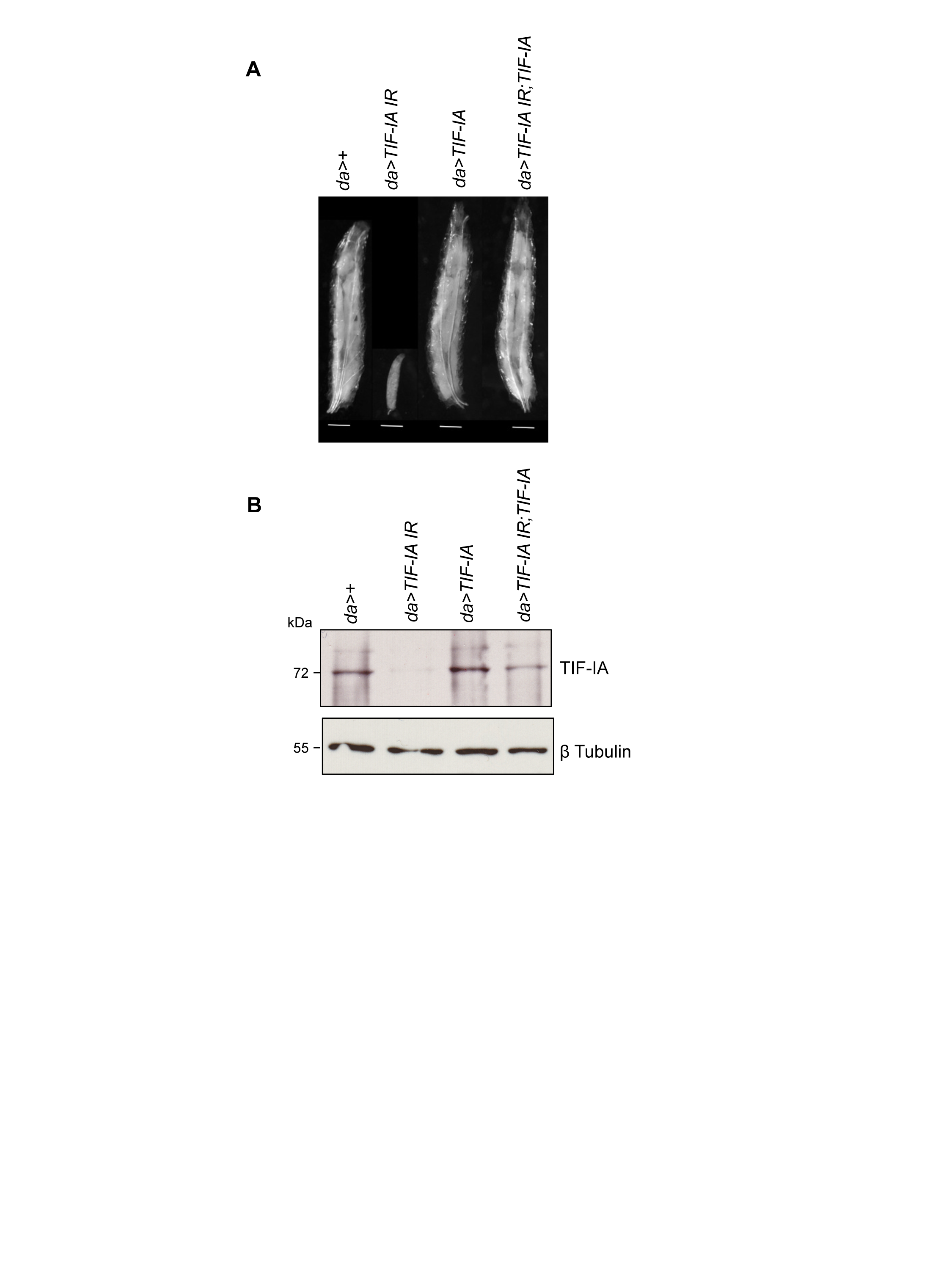

Supplement: Figure S1 — Co-expression of UAS-TIF-IA using da-GAL4 driver rescued the growth defects in da>TIF-IA IR larvae. (A) Representative images of 72 hr AEL larvae of indicated genotypes, scale bar-500 µm. (B) Immunoblot indicates TIF-IA protein levels in 72 hr AEL larvae of indicated genotypes. β tubulin levels indicate loading control. (TIF) [file pgen.1004750.s001.tif]

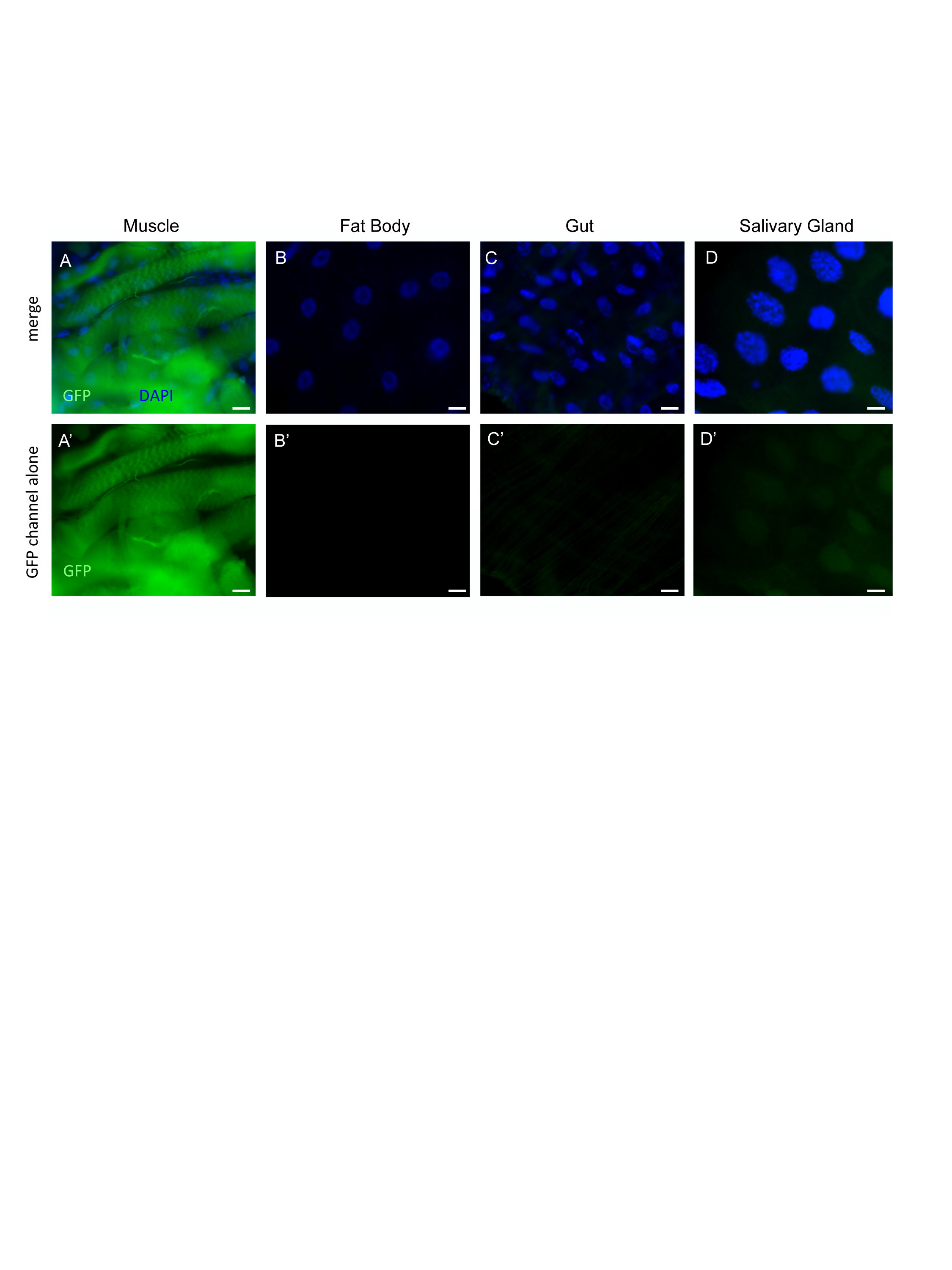

Supplement: Figure S2 — dMef2-GAL4 drives expression in body wall muscle. The dMef2-GAL4 was used to drive expression of UAS-GFP. Wandering larvae were fixed, dissected and mounted. Representative images of A, A′) body wall muscle, B, B′) fat body, C, C′) gut and D, D′) salivary gland are shown. All images were captured using the same exposure and magnification. (TIF) [file pgen.1004750.s002.tif]

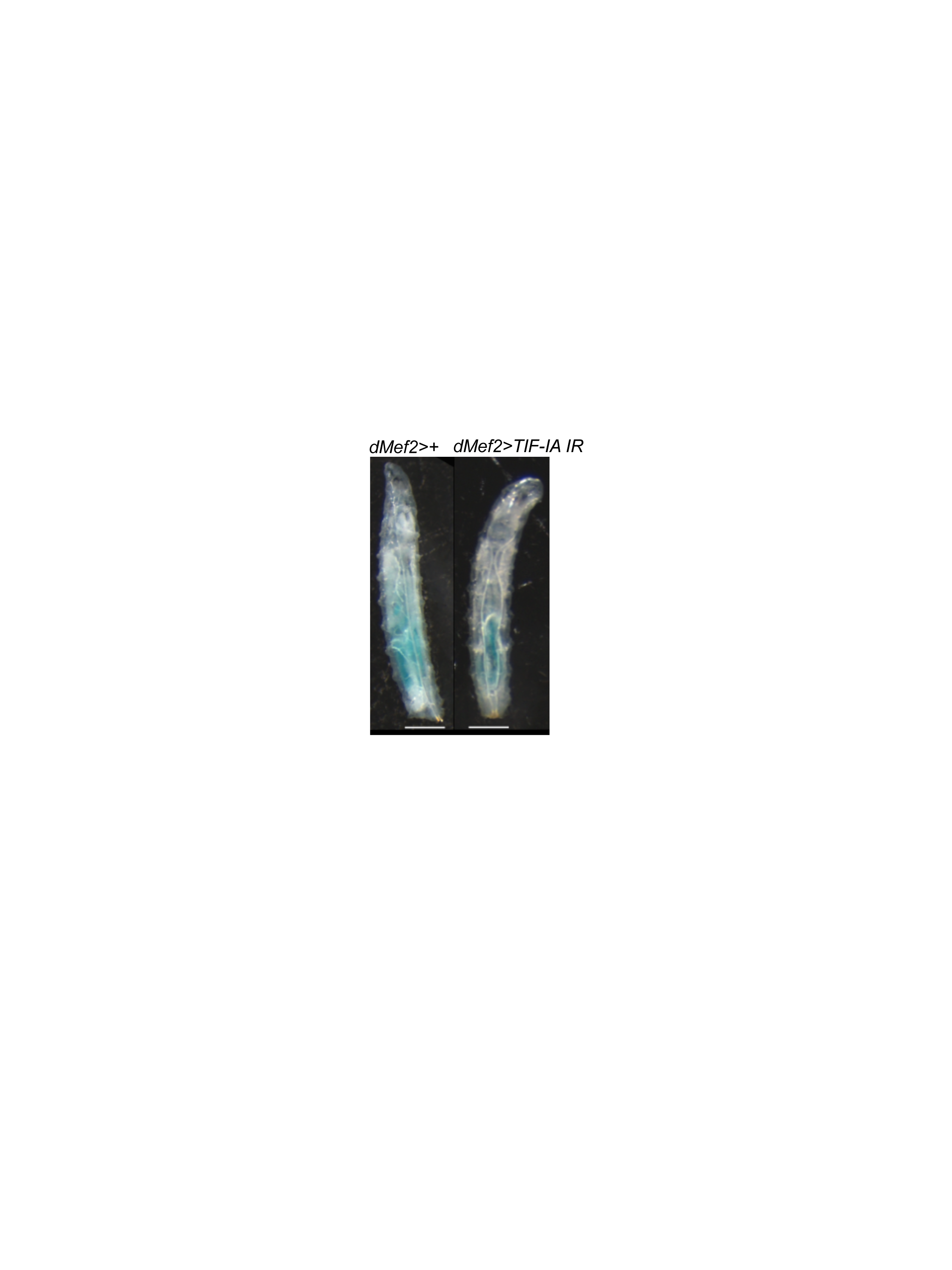

Supplement: Figure S3 — Muscle-specific TIF-IA inhibition does not affect larval food ingestion. Representative images of dMef2>+ and dMef2>TIF-IA IR larvae after 4 hrs of blue food (yeast paste colored with blue food dye) ingestion at 72 hr AEL, scale bar-500 µm. (TIF) [file pgen.1004750.s003.tif]

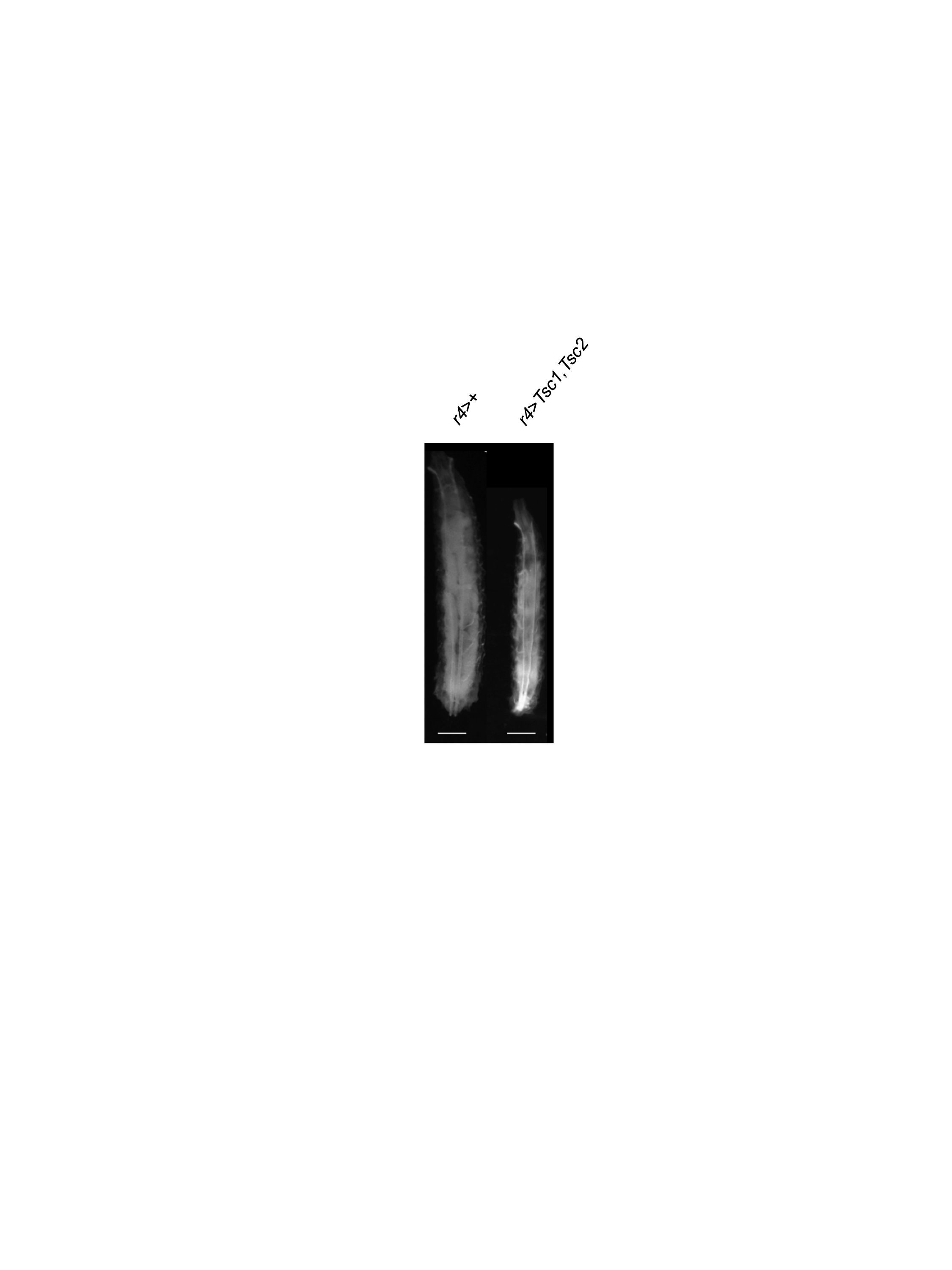

Supplement: Figure S4 — Overexpression of UAS-Tsc1 and UAS-Tsc2 in larval fat body inhibits body growth. Representative image of r4>+ and r4>Tsc1,Tsc2 larvae when r4>+ (control) larvae started wandering, scale bar-500 µm. (TIF) [file pgen.1004750.s004.tif]

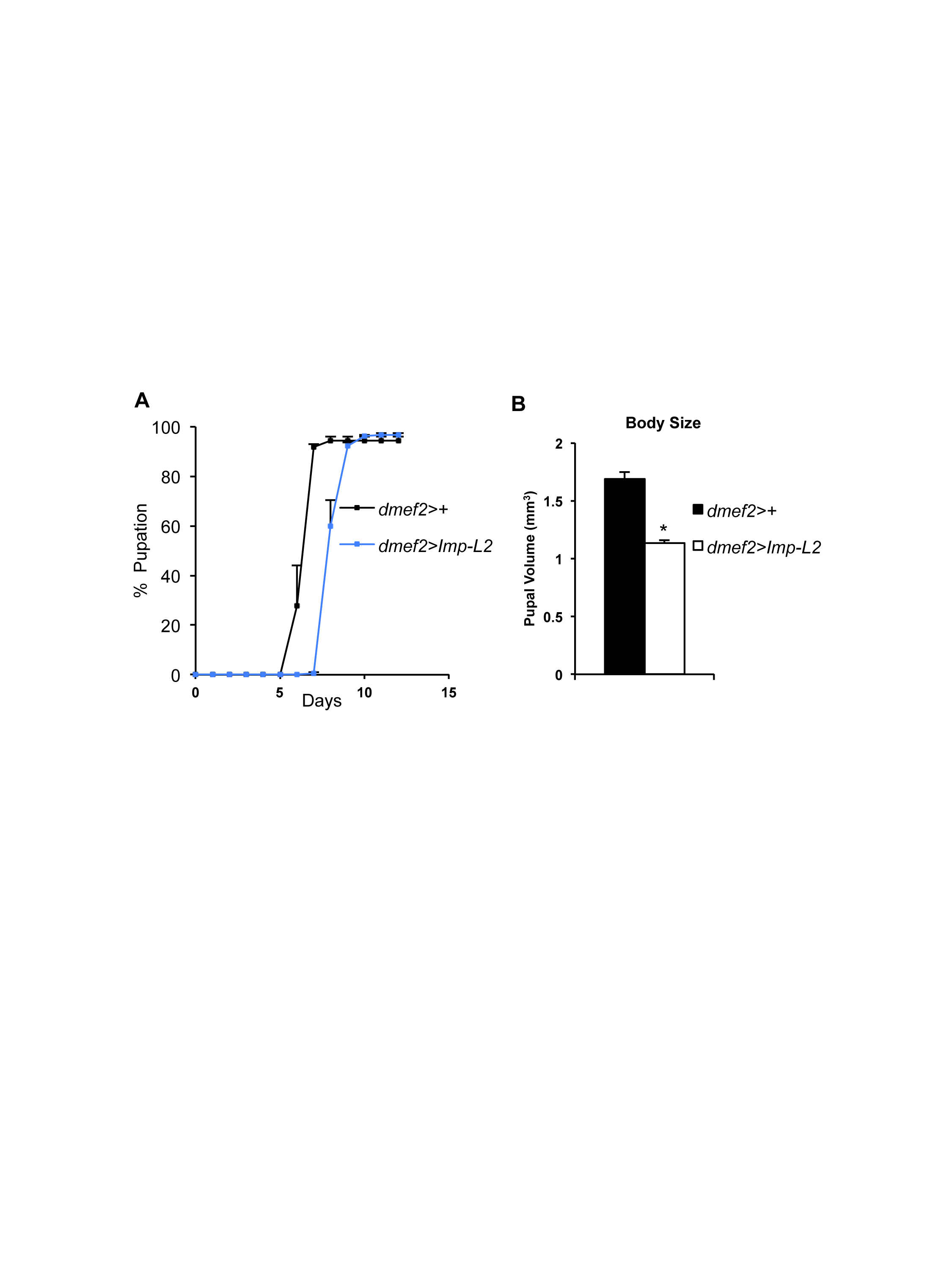

Supplement: Figure S5 — Overexpression of Imp-L2 in muscle delays development and inhibits body growth. (A) Developmental timing from larval hatching to pupation of dMef2>+ (n = 183) and dMef2>Imp-L2 (n = 180) animals, n - number of larvae assessed per genotype, mean time to pupation: dMef2>+, 6.79 days vs. dMef2>Imp-L2, 8.3 days, * P = 0.05, Mann-Whitney U test). (B) Pupal volume of dMef2>+ (n = 27) and dMef2>Imp-L2 (n = 32) pupae, n - number of pupae per genotype, (* P = 5.12×10^−11^, Student's t-test). (TIF) [file pgen.1004750.s005.tif]

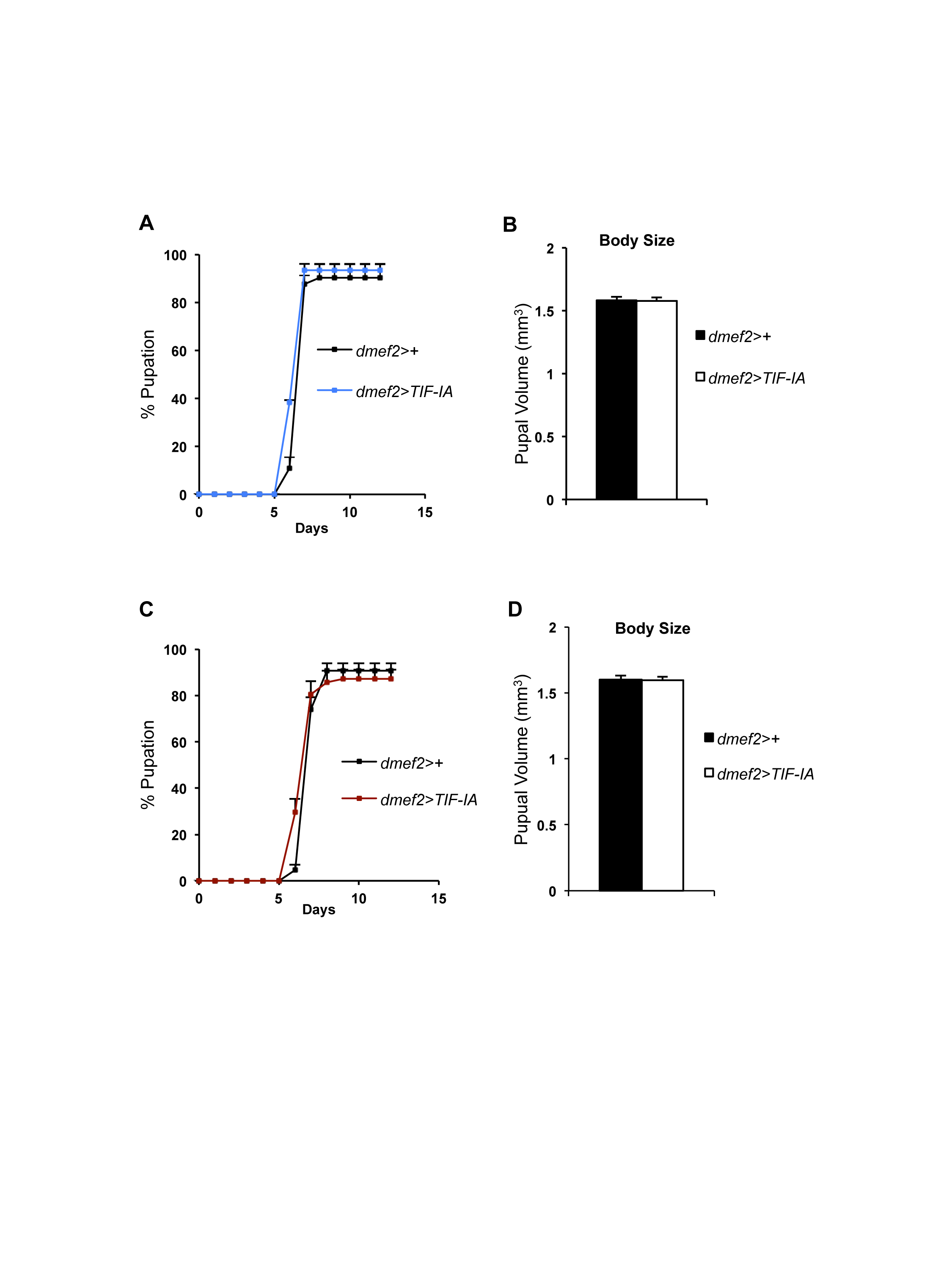

Supplement: Figure S6 — Overexpression of TIF-IA in muscle modestly accelerates development but does not promote body growth. (A) Developmental timing from larval hatching to pupation of dMef2>+ and dMef2>TIF-IA animals, n = 138, n - number of larvae assessed per genotype, (mean time to pupation: dMef2>+, 6.9 days vs. dMef2>TIF-IA, 6.6 days, * P = 0.05, Mann-Whitney U test). (B) Pupal volume of dMef2>+ (n = 101) and dMef2>TIF-IA (n = 102) pupae, n - number of pupae per genotype, (P = 0.88, Student's t-test). (C–D) Experiments were performed using a second UAS-TIF-IA transgene. (C) Developmental timing from larval hatching to pupation of dMef2>+ (n = 187) and dMef2>TIF-IA (n = 186) animals, n - number of larvae assessed per genotype, (mean time to pupation: dMef2>+, 7.1 days vs. dMef2>TIF-IA, 6.8 days, * P = 0.05, Mann-Whitney U test). (D) Pupal volume of dMef2>+ (n = 68) and dMef2>TIF-IA (n = 61) pupae, n - number of pupae per genotype, (P = 0.88, Student's t-test). (TIF) [file pgen.1004750.s006.tif]

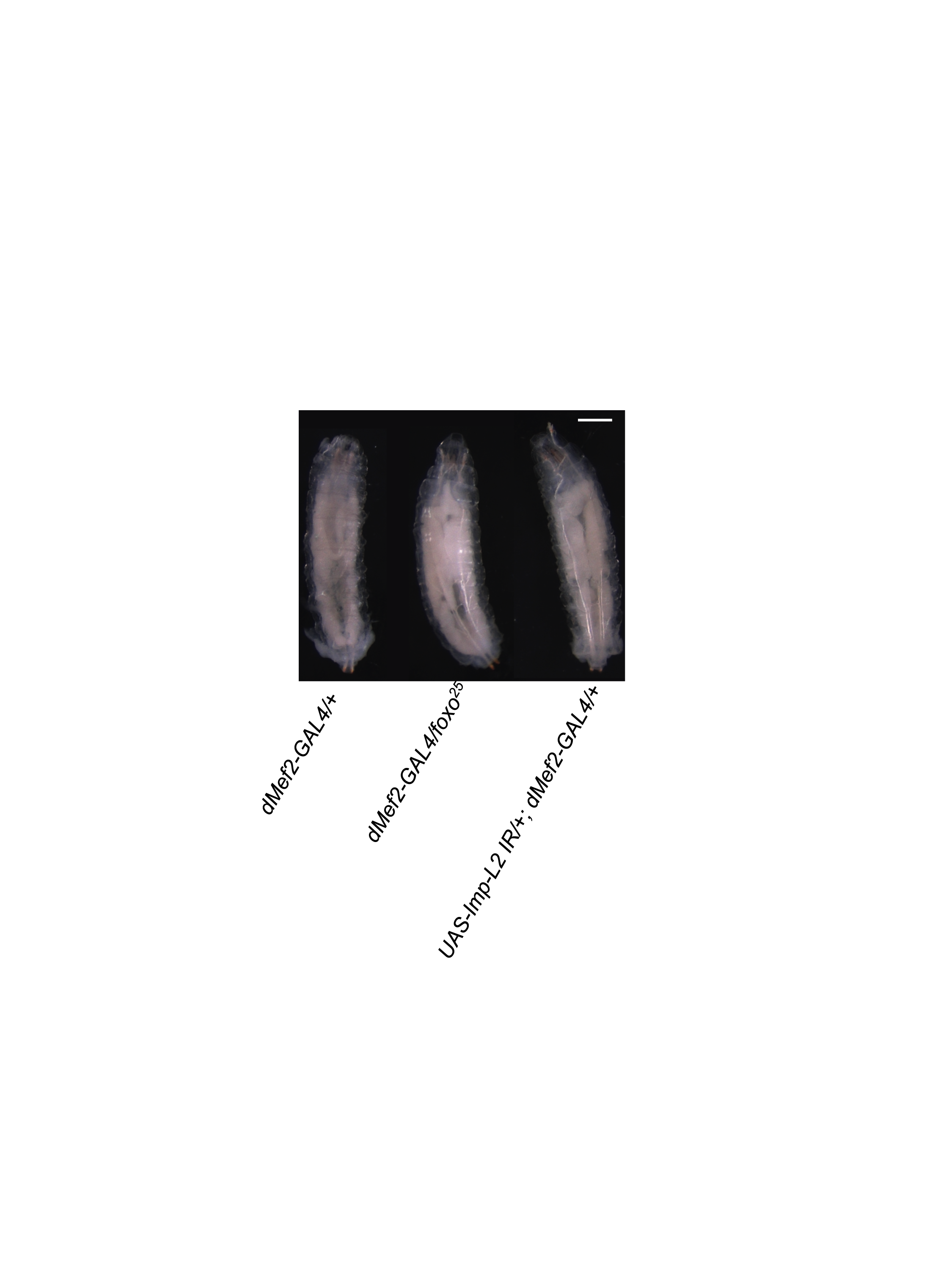

Supplement: Figure S7 — Loss of one copy of foxo or knockdown of Imp-L2 in muscle does not affect larval size. Representative images are shown of dMef2-GAL4/+ (left), dMef2/foxo25 (middle) and UAS-Imp-L2 IR/+; dMef2-GAL4/+ (right) larvae. All images were taken when the control (dMef2-GAL4/+) larvae were at the wandering L3 stage. Scale bar-500 µm. (TIF) [file pgen.1004750.s007.tif]
